# Divergent alternative mating tactics in convergent male reproductive morphs

**DOI:** 10.1093/beheco/araf086

**Published:** 2025-08-07

**Authors:** Renjie Zhang, Nathan W Bailey

**Affiliations:** School of Biology, University of St Andrews, St Andrews, Fife KY16 9TH, United Kingdom; School of Biology, University of St Andrews, St Andrews, Fife KY16 9TH, United Kingdom

**Keywords:** alternative reproductive tactic, behavioral plasticity, condition-dependent mating tactic, field cricket, satellite mating behavior, *Teleogryllus oceanicus*

## Abstract

Alternative reproductive phenotypes involve polymorphic behaviors and forms within sexes. Testing whether behavioral variants such as alternative tactics (eg sneaking or satellite behavior) are initially co-expressed or decoupled from morphological polymorphisms (eg weapon size or color pattern) can provide insight into the origins of reproductive diversity. In Hawaiian field crickets (*Teleogryllus oceanicus*), an eavesdropping parasitoid fly selected for rapid, parallel evolution of male wing mutations that reduce acoustic signals. Two of these, “flatwing” and “curly-wing”, co-occur in populations alongside ancestral “normal-wing” males that can sing. These convergent alternative morphs may both rely on satellite tactics in which nonsinging males position themselves near calling males to intercept females, rather than attracting mates directly by producing a conspicuous song. Here, we test whether flatwing and curly-wing vary in their tendencies to express satellite behavior using playback experiments with virgin, unmanipulated males simulating natural conditions. Surprisingly, flatwing males were significantly less likely to behave as satellites than normal-wing or curly-wing males. Normal-wing males with poorer body condition were more likely to behave as satellites, consistent with theory and previous findings, but the reduced-sound morphs showed no such condition dependence. Our findings suggest that morph-specific variation in the tendency to adopt satellite behavior may contribute to the maintenance of convergent male reproductive morphs; future work would benefit from testing whether such variation is driven by acoustic self-assessment. A decoupled relationship between behavioral reproductive tactics and morphological reproductive strategies may promote diversification of alternative mating morphs in nature.

## Teaser

We tested whether form and behaviour are coordinated when new mating strategies first evolve in nature. Hawaiian crickets recently evolved independent alternative wing forms: “flatwing” and “curly-wing” silence males and protect against eavesdropping predators. Unlike ancestral males, they cannot attract females by singing. These male forms are not equally likely to behave as a “satellite”, intercepting females responding to other males' songs. Instead, unique combinations of cricket form and behaviour have evolved.

## Introduction

Alternative reproductive phenotypes are thought to maximize reproductive success under varying, context-dependent selective pressures such as predation risk and mate competition; when genetically coded, they can be maintained by frequency-dependent selection (Ayala and Campbell 1974; Taborsky 1994; Andersson 1994; Gross 1996). Studies examining the evolution of polymorphic mating phenotypes focuses on two distinct properties. First, alternative reproductive *tactics* are behavioral polymorphisms within a single sex (eg sneaking or satellite behavior) that can be expressed due to condition dependence, plastic responses to the environment, or genotypic variation. Second, these varied behavioral tactics are often associated with morphological polymorphisms (eg weapon size or color pattern), termed alternative reproductive *strategies*. Such associations result in suites of traits that define roles such as guards versus sneakers in bluegill sunfish (*Lepomis macrochirus*), fighters versus sneakers in dung beetles (*Onthophagus acuminatus*) and callers versus satellites in acoustically signaling organisms such as anurans and field crickets (Cade 1979; Emlen 1997a; Fu et al. 2001; Shuster and Wade 2003; Leary et al. 2005). In some systems, more than two phenotypes exist, for example the blue, yellow, and orange forms of the side blotched lizard, *Uta stansburiana* (Gilman et al. 2019) or the independent, sneaker, and fader forms of the ruff, *Philomachus pugnax* (Küpper et al. 2016; Mank 2023). How these coordinated suites of behavioral and morphological characters originate evolutionarily is a topic of interest, with recent research emphasizing the detection of supergene architectures that cause co-inheritance of morphological and behavioral traits and depress recombination (Küpper et al. 2016). It is less appreciated, however, that alternative morphs of similar function can repeatedly and independently evolve and then co-occur in the same populations. Here, we examine whether independent alternative mating morphologies that evolve in parallel are, or are not, co-expressed with the same alternative behavioral tactics.

**Fig. 1.**
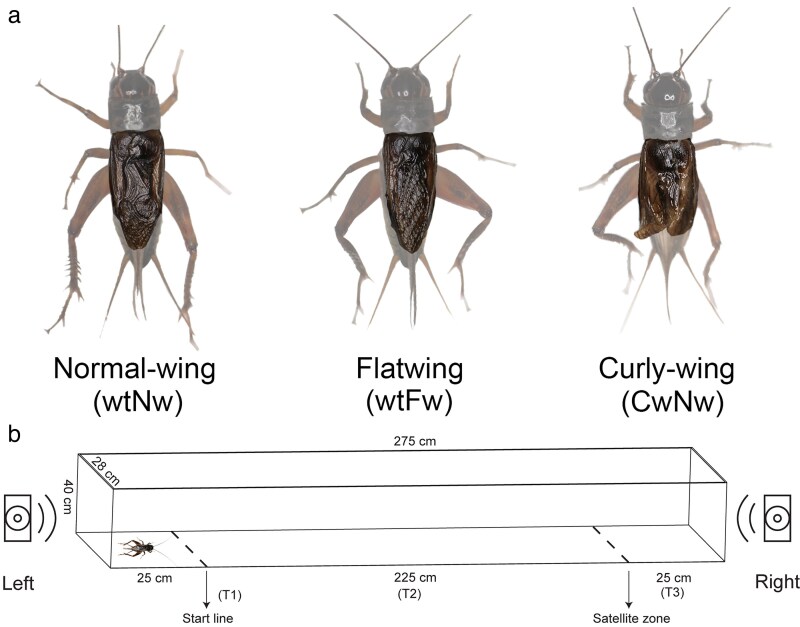
Morph description and experimental design. (a) Illustration of *T. oceanicus* wing morphs studied here: Wild-type normal-wing (wtNw) males have normal wing venation and undisrupted 3D wing structures; wild-type flatwing (wtFw) males lack specialized sound-producing wing veins and also have undisrupted 3D wing structures; curly-wing males can either express normal wing venation (CwNw), or flatwing venation (CwFw). This study only used the former, due to the rarity of CwFw males. Male forewings are highlighted. (b) Setup of phonotaxis trials. *T1* (*Response latency*) refers to the time when crickets crossed the start line after playback started. *T2* (*Response speed*) indicates the duration of time crickets spent moving towards the speaker after passing the start line. For ease of interpretation, this is reported in seconds as the distance was fixed. *T3* (*Satellite duration*) measures the duration crickets spent near the speaker. Only one speaker was active during each trial, and the position (left or right) of the functional speaker was alternated between trials. Photo credits: Renjie Zhang.

In Hawaiian populations of the field cricket *Teleogryllus oceanicus*, males exhibit a striking diversity of wing morphologies that were detected around two decades ago and which completely or partially silence their normal, acoustic mating calls (Zuk et al. 2006; Tinghitella et al. 2018; Rayner et al. 2019; Gallagher et al. 2022; Bailey et al. 2024; Zhang et al. 2024a). These alternative male morphs arose under competing selection pressures; firstly, to attract mates and secondly, to avoid predation by an eavesdropping parasitoid fly *Ormia ochracea* (Zuk et al. 2006; Tinghitella et al. 2018; Rayner et al. 2019; Gallagher et al. 2022, 2024; Zhang et al. 2024a). Calling behavior, although highly effective for attracting females, exposes males to lethal risks from these parasitoids (Cade 1975). Parallel evolution of signal loss occurred through the disruption of different physical structures of the sound-producing male forewing, and protects males from fly attack (Pascoal et al. 2014; Rayner et al. 2019, 2024; Zhang et al. 2021; Bailey et al. 2024). Several populations contain a mix of different, genetically determined male-silencing phenotypes. “Flatwing” and “curly-wing” both lack the ability to sing yet coexist alongside ancestral, “normal-wing” males that can sing (Fig. 1a) (Zuk et al. 2006; Rayner et al. 2019). Flatwing and curly-wing males are genetically determined male wing morphs with distinct genetic architectures but receive equivalent protection from parasitoids as neither can produce conspicuous mating calls (Zuk et al. 2006; Pascoal et al. 2014; Rayner et al. 2019, 2024). In some populations, neither phenotype has spread to fixation due to selective interference (Rayner et al. 2024).

We tested whether independently evolved alternative reproductive morphs in male crickets are associated with the same alternative behavioral tactic—satellite behavior, which is known to be expressed by both ancestral singing normal-wing males and silent flatwing males (Zuk et al. 2006; Tinghitella et al. 2009; Bailey et al. 2010; Olzer and Zuk 2018). Satellite behavior occurs when silent males position themselves near calling males to intercept approaching females, rather than attracting mates directly by producing a conspicuous signal (Cade 1979, 1980; Greenfield 1997). Satellite behavior is a common alternative mating tactic in taxa including birds (eg the ruff, *Philomachus pugnax*), mammals (eg topi, *Damaliscus lunatus*), anurans (toads and frogs), fish (eg *Porichthys notatus*), and invertebrates including field crickets (Wells 1977; Cade 1979; Gerhardt et al. 1987; Brantley et al. 1993; Leary et al. 2005). This strategy is often expressed by males in suboptimal condition, for whom the energetic costs of signaling or the risks of direct competition with stronger males outweigh the potential benefits (Leary et al. 2005, 2024; Bailey et al. 2010). Moreover, adopting a satellite strategy significantly reduces the risk of parasitism in the few hosts of *O. ochracea*. For example, the parasitism rate has been found to decrease from 82% in calling males to only 6% in satellite males in *Gryllus integer* (Cade 1975). In Hawaiian *T. oceanicus*, the satellite strategy is a prominent, but not exclusive, mechanism by which flatwing crickets secure mating success (Zuk et al. 2006; Schneider et al. 2024). However, it is not clear whether the propensity to express this pre-established mating tactic varies among newly evolved silent morphs, and whether condition dependence of the strategy has been lost in these morphs. Determining how consistently—or variably—the behavioral and morphological components of alternative reproductive phenotypes are co-expressed can help to dissect the relative roles of behavioral and morphological innovation in the evolution and maintenance of polymorphic mating strategies.

We focus on variation in the expression of satellite tactics—the tendency to express satellite behavior, as well as response latency, speed, and the expected negative association of satellite behavior with body condition—among recently evolved silent *T. oceanicus* morphs. We focus on flatwing and curly-wing morphotypes as they are caused by different genetic mutations, differ in morphology and signal reduction, but have the same functional outcome of sexual signal loss-of-function (Rayner et al. 2024). To mimic natural conditions, we did not manipulate males’ ability to produce and perceive sound, and reared them together under the same acoustic background. Our general hypothesis is that behavioral trait components of alternative mating tactics evolve separately from morphological components of alternative mating tactics—here, satellite behavior and the mutations affecting wing structures that generate sound, respectively. As a useful contrast to evaluate any variability in satellite tendencies of flatwing versus curly-wing males, we compare their satellite behavior to that of the normal-wing male morph, which is known to be the ancestral state in this system (Bailey et al. 2024) and which is known to exhibit condition-dependent satellite behavior (Bailey et al. 2010).

Prior information motivates predictions about morph differences in satellite behavior. There has been rapid evolution of locomotion and sensory processing in natural populations of flatwing crickets (Schneider et al. 2024; Zhang et al. 2024b), and normal-wing males can attract mates using song and have been found repeatedly in field studies to be less likely to exhibit satellite behavior than flatwing morphs (Zuk et al. 2006, 2018). Thus, we anticipated that normal-wing males would be the least likely to employ satellite mating tactics. In several wild populations, the *curly-wing* mutation invaded after the *flatwing* mutation but quickly reached a high abundance (Rayner et al. 2024). This suggests a competitive advantage that might be mediated by a greater tendency to adopt satellite mating tactics. By this logic, we anticipated the following order of increasing satellite tendency: normal-wing < flatwing < curly-wing. However, normal-wing males can readily hear their own song and thus have an available metric to evaluate their own quality, curly-wing males occasionally produce some low-intensity sounds, and flatwing males are completely silent. If feedback about one's own ability to attract mates by singing dominates individual decisions to behave as a satellite, then we would anticipate the following order of increasing satellite tendency: normal-wing < curly-wing < flatwing. Surprisingly, we found that flatwing males were less likely to adopt satellite tactics than the other two morphs, while there was no significant difference between curly-wing and normal-wing males. Coupled with variation in condition dependence of satellite behavior across the morphs, these findings suggest that noncoordinated evolution between the morphological and behavioral components of alternative reproductive tactics can contribute to the maintenance of multiple, novel morphological adaptations in natural populations, a situation that is observed in many systems such as blue-tailed damselflies, side-blotched lizards, freshwater fish, and guppies (Gilman et al. 2019; Sandkam et al. 2021; Mank 2023).

## Materials and methods

### Cricket origins and rearing

We used *T. oceanicus* laboratory stock originally derived from a population at a Community Center in Manoa in 2017 (Lat: 21.316219, Long: −157.809922), on the Hawaiian island of Oahu, which is the first documented population harboring audible normal-wing males with adaptive silent curly-wing and flatwing males (Rayner et al. 2019). Crickets were kept at a population size of approximately 100 breeding individuals, reared at a temperature of 25 °C under a 12:12 h light:dark cycle. Rabbit chow (Burgess Excel Junior and Dwarf rabbit pellets) and water were provided ad libitum, and cardboard egg cartons were provided for shelter. Maintenance was performed twice weekly.

In the last instar stage, male nymphs were manually separated and individually housed in 113 ml transparent plastic deli cups (height: 3.8 cm, lower diameter: 5.4 cm, upper diameter: 7.0 cm) containing a small amount of rabbit chow, a water bottle with cotton, and a single piece of egg carton. We checked the isolated individuals for eclosion every 2 days. Upon eclosion, crickets were phenotyped by visual inspection. We pooled individual deli pots of same morph into 14-L open plastic boxes for transportation. All crickets were then housed in the same incubator with their positions randomized after each maintenance session. This ensured that all subjects experienced a uniform ambient acoustic environment during rearing. Because individuals were maintained separately in deli pots, we were able to accurately track their eclosion dates.

### Phonotaxis trials

Phonotaxis trials were modified from protocols established by Bailey et al. (2010) and Bailey and Macleod (2014). Trials were conducted in a soundproof room at 25 ± 1 °C under red light during the crickets’ dark cycle. Each cricket was placed in a rectangular wooden box (275 × 28 × 40 cm) with foam-lined sides to minimize echoes. The apparatus had a Logitech Z120 speaker positioned at each end, and song playback was controlled via a SanDisk MP3 player (SDMX26-008G-E46P) connected to the speakers. Only one speaker played sound during each trial, allowing playback direction to be randomized. Speakers were placed directly behind plastic mesh-covered rectangular openings (10 cm in length × 6 cm in width) on both ends of the chamber to prevent subjects from crawling behind or underneath the speakers. Figure 1b shows a diagram of the experimental arena.

During each trial, only one speaker played a randomly selected song model and the test cricket was placed at the opposite end from the active speaker. The song model and active speaker were switched between trials. We used four *T. oceanicus* calling songs recorded from normal-wing males from laboratory stock of the same population for use as playback songs. As described previously (Rayner et al. 2019), songs were recorded at a distance of 30 cm from males using a Sennheiser ME66 microphone at 25 ± 1 °C and then looped into 10-minute.mp3 files, each containing 3 to 4 complete song phrases. Carrier frequency (the frequency with the highest energy in the signal) of the song models were measured from each unlooped recording using the “Plot Spectrum” function in Audacity v.3.3.2 (Table S1). Additional song parameters are provided in Table S1, and all song models are available as supplementary files (Supplementary audio files S1 to S4). During experiments, we played songs back at a peak amplitude of approximately 70 dB SPL measured 50 cm from the speaker using a digital sound-level meter (CEM-DT-805, Shenzhen, China), simulating the intensity of a male song in the field (Simmons et al. 2001). Males aged 5 to 8 days post-adult eclosion were used in phonotaxis trials. Individuals at this age are sexually mature and exhibit robust phonotactic responses (eg Bailey et al. 2010). Younger individuals (<5 d-old) may not yet be fully sexually receptive, while older individuals (>10 d-old) may show reduced responsiveness due to aging or decreased mating motivation (Sarmiento-Ponce et al. 2021). Using crickets within this age range (5 to 8 d-old) helps minimize variability associated with age-related physiological or motivational differences. Each subject was transferred within its deli pot to the inside of a soundproof foam cell for at least 30 min prior to testing, to eliminate any recent acoustic exposure. The subject was then placed at the end of the chamber opposite the active speaker and allowed to acclimate under an upside-down deli cup in silence for 2 min. At the start of playback, the deli cup was removed gently and the trial was timed with a stopwatch. Each trial lasted 5 min. Odor cues were eliminated after each trial by cleaning with 70% ethanol.

Two vertical lines were marked 25 cm from each end of the chamber to designate the starting line and the boundary of the satellite zone (Fig. 1b). Then, we recorded the following metrics: (1) Whether or not the male responded to the playback by entering the 25 × 28 cm rectangular area directly in front of the speaker, ie the “satellite zone”, as such movement toward a conspecific signal without subsequent song or display is a well-established proxy for satellite behavior (Cade 1980). For those that approached the satellite zone, we recorded: (2) *Response latency* as the time taken to cross the starting line, (3) *Response speed* as the time taken for the male to move from the starting line to the satellite zone in front of the speaker, (4) *Satellite duration* as the total time the male spent inside the satellite zone (multiple entries were cumulative).

After trials, we measured each male's pronotum length to the nearest 0.01 mm using a digital Vernier caliper (RTP 6″, Rowland Tools). Three independent measurements were averaged to obtain a final value. Body mass was weighed to the nearest 0.001 g using a digital balance (BDH AL-300). To quantify body condition, we then calculated the scaled mass index (SMI) using the averaged pronotum length and body mass for subsequent analyses, following Peig and Green (2009). R code used for these calculations was adapted from Rayner et al. (2024).

### Analyses

Statistical analyses were conducted in R v.4.3.0 (R Core Team 2023) and data visualization was performed using the **ggplot2** package (Wickham 2016). Prior to analyses, data distributions were evaluated using residual diagnostics, including Q–Q plots and density plots generated using the **ggpubr** package (Kassambara 2023). Normality was assessed with Shapiro–Wilk tests, while Levene's tests were used to examine the homogeneity of variances in SMI among all tested male morphs, and between satellite and nonsatellite males.

Factors associated with the expression of satellite behavior in males were analyzed using generalized linear models (GLMs) with a binomial distribution to assess overall effects, including predictors such as phenotype, SMI, speaker side, song models, and the interaction between phenotype and SMI. We then performed post hoc pairwise contrasts using Fisher's Exact tests to assess whether morphs differed in their response to model male calling songs, given the small sample size for satellite flatwing males. We used ANOVA test to compare SMI among male morphs. To compare *response latency*, *response speed*, and *satellite duration*, separate GLMs with negative binomial distributions were fitted using the **MASS** package (Venables and Ripley 2002), as severe overdispersion was detected using the DHARMa package (Hartig 2022). Significance was determined using chi-square tests with Type II sums of squares, as implemented in the **car** package (Fox and Weisberg 2019). McFadden's pseudo *R*^2^ was calculated using the “nagelkerke” function from the **rcompanion** package (Mangiafico 2023). Due to missing data at some of the time points, 4 samples were excluded from these analyses (1 wtNw, 2 wtFw, and 1 CwNw). Two-tailed t-tests were performed as a post hoc test to compare differences in body condition between satellite and nonsatellite males within each morph. We used Spearman correlation tests to examine the associations between satellite duration and response latency, and between satellite duration and response speed across all males who expressed satellite behavior.

## Results

### Expression of satellite behavior by parallel male morphotypes

**Fig. 2.**
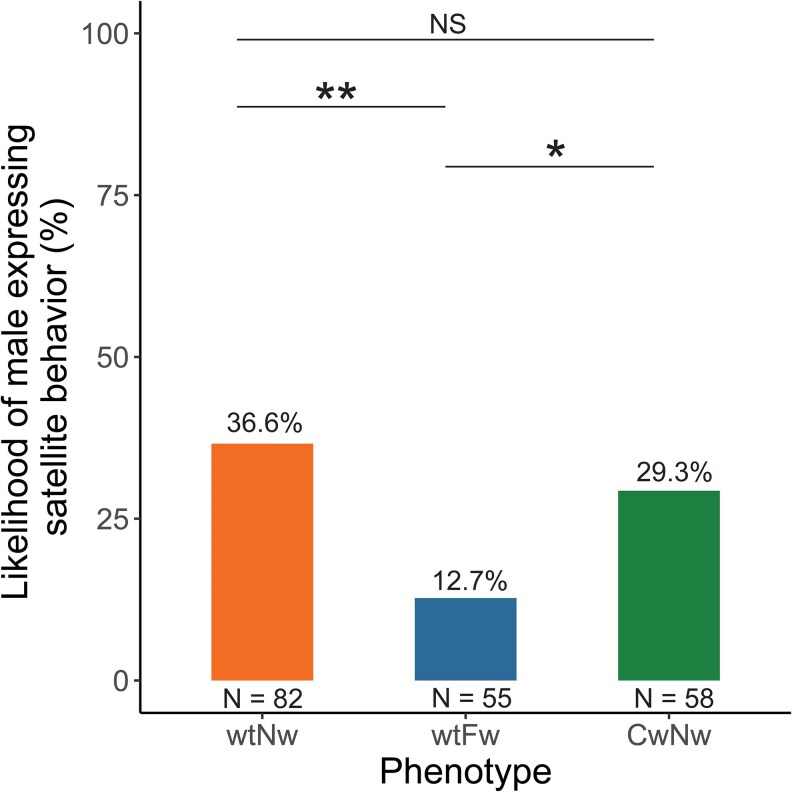
Morph differences in expression of satellite behavior. Likelihood of males expressing satellite tactics during a 5-min phonotaxis trial. *P*-values were calculated using Fisher's exact tests. Asterisks indicate statistical significance (*P < 0.05, **P < 0.01). NS, not significant. See main text for details.

Satellite behavior was monitored in 195 males (normal-wing: 82, flatwing: 55, curly-wing: 58). Phonotaxis trials revealed that all three male morphs adopt the satellite strategy, but vary in their tendency to do so. Normal-wing males were the most likely to express satellite behavior (36.6%), followed by curly-wing males (29.3%), and flatwing males (12.7%) (Fig. 2). The likelihood of satellite behavior was significantly depressed for flatwing males compared to the other two morphotypes (Fisher's exact tests: normal-wing vs. flatwing: *N* = 137, *P* = 0.003, curly-wing vs. flatwing: *N* = 113, *P* = 0.039). There was no difference between normal-wing and curly-wing males (Fisher's exact test: normal-wing vs. curly-wing: *N* = 140, *P* = 0.47). We found no effect of song models or the direction in which the speakers were positioned (Table 1).

**Table 1.** GLM examining factors that predict the likelihood of male satellite behavior (*N* = 195, *R*^2^ = 0.086).

Predictor	*df*	*χ* ^2^	*P*
**Phenotype**	2	9.8008	**0.007**
**SMI**	1	0.9735	0.324
**Side of playback**	1	0.0355	0.851
**Song model**	3	1.3342	0.721
**Phenotype:SMI**	2	7.0315	**0.030**

Significant *P*-value (<0.01) is highlighted in bold.

### Body condition and satellite behavior

**Fig. 3.**
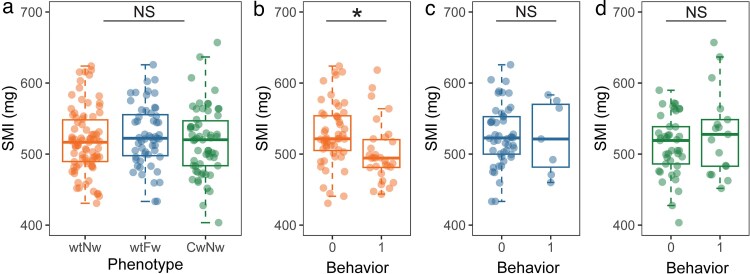
Post hoc contrasts assessing the relationship between condition and satellite behavior. (a) Comparison of SMI across morphs. (b to d) Comparison of SMI between nonsatellite (“0”) and satellite (“1”) males within normal-wing, flatwing, and curly-wing morphs, respectively. Medians (solid lines), inter-quartile ranges (boxes), and 1.5 × inter-quartile range (whiskers) are shown inset with data points jittered for clarity. *P*-values were calculated using ANOVA and t-tests. Asterisks indicate statistical significance (*P* < 0.05). NS, not significant. See Main text for statistical details.

Morph-specific analyses revealed no statistically significant difference in condition among morphs (Fig. 3a) (ANOVA: F_2,192_ = 0.571, *P* = 0.566). However, a significant morph*SMI interaction for satellite behavior indicated that the effect of body condition on expression of satellite behavior varied among male phenotypes (*P* = 0.030; Table 1). Specifically, *post-hoc* analysis revealed that among normal-wing males, lower-condition individuals approached the playback more often than higher-condition males (Fig. 3b) (post hoc t-test: *t* = 2.560, df = 80, *P* = 0.01). This effect was absent in the two silent morphs, with no differences in body size detected between satellite and nonsatellite males (Fig. 3c and d) (post hoc t-tests: curly-wing, *t* = −1.168, df = 56, *P* = 0.25; flatwing, *t* = 0.142, df = 53, *P* = 0.89). Note that data related to flatwing satellite males should be interpreted with caution, due to the small number of individuals displaying satellite behavior during trials.

### Latency, speed, and duration of satellite responses

**Fig. 4.**
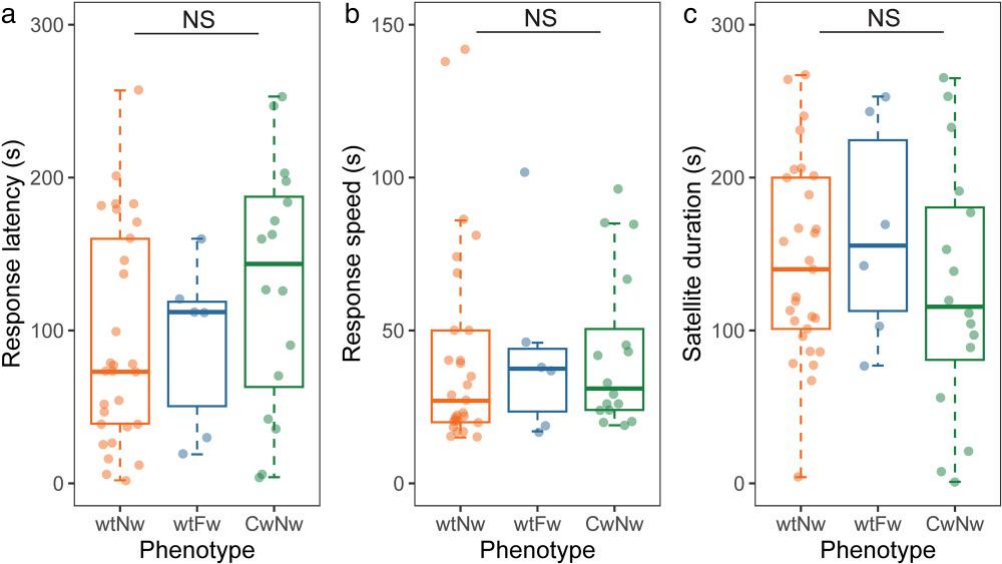
Timing of satellite responses across morphs. (a) Response latency. (b) Response speed. (c) Satellite duration. Medians (solid lines), inter-quartile ranges (boxes), and 1.5 × inter-quartile range (whiskers) are shown inset with data points jittered for clarity. *P*-values were obtained from GLMs. NS, not significant. See Main text for statistical details.

Among those males that did adopt satellite tactics, we found no significant variation in response time to playbacks across the three morphs (Fig. 4a; Table 2). There was also no evidence of any morph difference in speed when moving towards to the playback (Fig. 4b; Table 2). Lastly, no morph tended to spend more time in the satellite area (within 25 cm of the speaker) compared to the others (Fig. 3c; Table 2). SMI significantly predicted response speed but not response latency or satellite duration (Table 2). A side of playback effect for response speed (Table 2) potentially reflects asymmetries in the acoustics of the experimental arena or lateralized behavioral tendencies. Significant positive correlations between satellite duration and response latency (Spearman rank correlation, *N* = 51, *r* = −0.805, *P* < 0.001), and between response speed and satellite duration (Spearman rank correlation, *N* = 51, *r* = −0.337, *P* = 0.016) in the pooled dataset provide confidence that our distinct measures of satellite behavior broadly capture the same underlying behavioral phenomenon.

**Table 2.** GLM examining timing of satellite behavior across male phenotypes.

Timing	Predictor	*df*	*χ* ^2^	*P*
***Response latency*** ***N* = 51,** ***R* ^2^ = 0.004**	Phenotype	2	1.9301	0.381
	SMI	1	0.2430	0.622
	Side of playback	1	0.1759	0.675
	Song model	3	0.1213	0.989
***Response speed*** ***N* = 51,** ***R* ^2^ = 0.025**	Phenotype	2	1.4540	0.483
	SMI	1	4.6168	**0.032**
	Side of playback	1	8.0625	**0.005**
	Song model	3	3.0410	0.385
***Satellite duration*** ***N* = 51,** ***R* ^2^ = 0.005**	Phenotype	2	0.5048	0.777
	SMI	1	0.9734	0.324
	Side of playback	1	0.7378	0.390
	Song model	3	0.9378	0.816

Significant *P*-values (<0.05) are highlighted in bold.

## Discussion

Alternative reproductive phenotypes are widely observed across the animal kingdom and contribute to diversity in mating systems (Wells 1977; Cade 1979; Gerhardt et al. 1987; Brantley et al. 1993; Emlen 1997a; Mank 2023; Leary et al. 2024). Flexible mate acquisition or courtship behaviors allow morphologically divergent males to react to their environmental and social conditions, such as competition intensity or predation risk, in a way that reduces direct competition with other morphs and maximizes fitness. Different combinations of alternative reproductive morphologies and behavioral mating tactics may facilitate the persistence of multiple reproductive strategies and thus phenotypic diversity within populations.

The relative lack of satellite behavior by flatwing males was surprising and contrary to our predictions. Instead, curly-wing and normal-wing males were both more likely to adopt satellite behavior. This outcome contrasts with previous findings from field studies (Zuk et al. 2006, 2018), which reported that flatwing males more frequently adopted satellite behavior to compensate for their lack of acoustic signaling, and a more recent laboratory study reporting no evidence that flatwing and normal-wing males differ in the likelihood of satellite behavior (Olzer and Zuk 2018). Methodological differences might explain this difference and shed light on the dynamics of alternative mating tactics in this system. Olzer and Zuk (2018) used a laboratory population established prior to the emergence of convergent male-silencing morphs including curly-wing; it is plausible that evolutionary interactions involving both silent morphs and normal-wing males affect the relative success of different behavior-morphology combinations for males, for example via negative frequency-dependent selection (Ayala and Campbell 1974; Andersson 1994; Taborsky 1994; Gross 1996). Second, selection pressures shaping behavioral strategies are likely to differ across populations and over time. Flatwing males studied in the wild are likely to have achieved fewer mattings than normal-wing males owing to their silence, and the environment that they experience may largely lack singing males. The experience of a silent acoustic environment enhances adoption of satellite tactics (Bailey et al. 2010) and increases locomotion (Balenger and Zuk 2015; Schneider et al. 2024). Thus, demographic factors may enhance flatwing males’ satellite tendencies in natural conditions compared with controlled laboratory conditions where all subjects have the same prior mating experience (ie are virgin) and are in the most active stage of sexual maturity.

Most research examining the decision to adopt alternative reproductive behavioral tactics focuses on condition dependence, and male size is often associated with shifts in mating tactics. For example, poorer-condition males of many insect species tend to adopt satellite strategies to avoid direct mate competition (Emlen 1997b; Hunt and Simmons 2001; Tomkins et al. 2004; Tomkins and Brown 2004; Radwan 2009; Walling et al. 2009; Wilgers et al. 2009; Bailey et al. 2010). However, less is known about other aspects of male condition that may contribute to transitions from one mating tactic to another. Here, we observed that body size influenced satellite behavior only in normal-wing males, who have the capacity to evaluate themselves by perceiving their own signals. In contrast, no such effect was detected in silent morphs, who lack the instantaneous acoustic feedback that can be used to assess their attractiveness. Given the known association of calling song parameters with body size in crickets (Ryder and Siva–Jothy 2000), the possibility and mechanisms of self-assessment based on self-generated song warrants further experimental investigation. While we cannot definitively demonstrate self-evaluation without neurophysiological data, this pattern suggests that the loss of feedback from acoustic signals may disrupt the mechanisms underpinning self-assessment, leading silent morphs to rely more heavily on environmental cues when adopting satellite tactics. It is possible that a capacity for self-assessment has been evolutionarily lost in flatwing males, though this would require the genetic decoupling of a complex behavioral feedback mechanism from the causative flatwing locus due to relaxed selection. If feedback from signaling interacts with body condition to influence behavioral decisions of flatwing males, it is worth considering whether they experience increased selection to shift to visual or chemical signaling for mate attraction to overcome this penalty, as suggested in other insects (Candolin 2003; Johansson and Jones 2007; Stoffer and Walker 2012; Gray et al. 2014; Assis et al. 2017).

Investigating other traits related to mating behavior, such as the neural and physiological basis of behavioral plasticity underlying alternative reproductive phenotypes, could provide insights on how genetic and phenotypic variation are maintained in natural populations. Key to such investigation is determining genetic correlations between morphological and behavioral components of alternative reproductive phenotypes. Satellite behavior is heritable in other cricket species (eg *G. integer*; Cade 1981). While it shows a clear signature of condition dependence in this and other cricket species (Wagner Jr and Hoback 1999; Scheuber et al. 2003; Bailey et al. 2010), the contribution of genetic versus environmental factors in determining the expression of satellite behavior is unknown in *T. oceanicus*, and there may be genetic variation in environmental switchpoints as has been observed in other systems (eg forceps size in the earwig *Forficula Auricularia*; Tomkins and Brown 2004). Such variation would provide a genetic substrate upon which selection may act to link morphological and behavioral reproductive traits. In some classic systems such as the ruff and white-throated sparrows, such linkage arises due to inversion/supergene architectures (Küpper et al. 2016; Tuttle et al. 2016), which stabilize co-adapted suites of behavioral and morphological traits but constrain the potential for further diversification beyond the initial morphs established by the genetic linkage. In contrast, independent genetic architectures could release constraints on the diversification of intrasexual polymorphisms, allowing traits to evolve independently and potentially favor the emergence of novel trait combinations. Our findings in *T. oceanicus* male-silencing morphs suggest the latter scenario is more likely in this system. Further exploration of the genetic correlations of morphological, behavioral, neural, and physiological traits in this system could enhance our understanding of how phenotype and behavior are evolutionarily co-opted or reciprocally inhibited in response to different selective pressures.

## Supplementary Material

araf086_Supplementary_Data

## Data Availability

Analyses reported in this article can be reproduced using the data provided by (DOI: https://doi.org/10.5061/dryad.x95x69pxf).
