# Supplementary material for: Divergent alternative mating tactics in convergent male reproductive morphs
Source: Behav Ecol. 2025 Aug 7;36(5):araf086. doi: 10.1093/beheco/araf086 (PMC12449065; doi:10.1093/beheco/araf086)
Supplement: araf086_Supplementary_Data [file araf086_supplementary_data.zip › Supplementary Table 1.docx]

**Supplementary Table 1.** Parameters of song models used in phonotaxis trials.

| **Song model** | **Carrier frequency (kHz)** | **Numbers of songs per unit** | **Duration (s)** | | **Interval between**  **song unit (s)** | **Numbers of song unit** |
| --- | --- | --- | --- | --- | --- | --- |
| A | 4.847 | 3 | | 2.66 | 0.03 | 112 |
| B | 4.918 | 3 | | 4.08 | 0.04 | 73 |
| C | 5.115 | 3 | | 4.37 | 0.05 | 68 |
| D | 4.735 | 4 | | 4.66 | 0.08 | 64 |
